# Multimodality imaging differentiation of pancreatic neuroendocrine tumors and solid pseudopapillary tumors with a nomogram model: A large single-center study

**DOI:** 10.3389/fsurg.2022.970178

**Published:** 2022-10-06

**Authors:** Hai-Feng Hu, Zheng Li, Ke Chen, Meng-Qi Liu, Zeng Ye, Xue-min Chen, Yue Zhang, Xian-Jun Yu, Xiao-Wu Xu, Shun-Rong Ji

**Affiliations:** ^1^ Center for Neuroendocrine Tumors, Fudan University Shanghai Cancer Center, Shanghai, China; ^2^ Department of Pancreatic Surgery, Fudan University Shanghai Cancer Center, Shanghai, China; ^3^ Department of Oncology, Shanghai Medical College, Fudan University, Shanghai, China; ^4^ Shanghai Pancreatic Cancer Institute, Shanghai, China; ^5^ Pancreatic Cancer Institute, Fudan University, Shanghai, China; ^6^ Department of Endoscopy, Fudan University Shanghai Cancer Center, Shanghai, China; ^7^ Department of Hepatopancreatobiliary Surgery, The Third Affiliated Hospital of Soochow University, Changzhou, China

**Keywords:** pancreas, pancreatic neuroendocrine tumor, solid pseudopapillary tumor, multimodality imaging, clinical differentiation

## Abstract

**Background:**

Pancreatic neuroendocrine tumors (pNETs) and solid pseudopapillary tumors (SPTs) are two of the most common pancreatic neoplasms with different treatment procedures. However, the broad heterogeneity of pNETs and SPTs in clinical manifestations and radiological features often confuse the presurgical discrimination in clinical practice, and the clinical and molecular differentiation of the two tumors remains elusive to date. We presume that a large and comprehensive study into the multimodality features of pNETs and SPTs is necessary for precise clinical management.

**Methods:**

We collected and analyzed the clinicopathological information and multimodality features of nonfunctional pNET and SPT patients, for a total of 631 cases from 2006 to 2021. Univariate analysis of imaging features, including contrast-enhanced computed tomography (CT), magnetic resonance imaging, endoscopic ultrasound (EUS) and nuclear medicine imaging, and clinical characteristics was performed, and CT features and clinical information were integrated to establish a nomogram model.

**Results:**

We recruited 354 nonfunctional pNET and 277 SPT patients in our cohort. Regarding demographic information, pNET patients had a lower female percentage (55.4% vs. 72.9%), smaller tumor size (2.8 vs. 4.8 cm), and older age (53.4 vs. 35.3 years). In CT imaging and EUS, pNETs tended to appear as solid and homogenous lesions with strong enhancement intensity. Multifocal lesions, duct dilation, and lymph node (LN) enlargement were more likely to be observed in pNETs, while calcification was more common in SPT lesions. On positron emission tomography (PET)/CT, pNETs exhibited significant sensitivity to somatostatin receptor scintigraphy (SRS), with positive rates of 81.4% and 95% on ^99^mTc-HYNIC-TOC and ^68^Ga-DOTATATE PET/CT, respectively, while SPTs were all negative on SRS. Multivariate analysis identifies tumor size, age, enhancement intensity, calcification, and LN enlargement as statistically significant variables.

**Conclusions:**

Compared to SPT patients, pNET patients exhibit an older age and smaller tumor size. CT manifestations of strong intensity, LN enlargement, and no calcification could indicate a higher possibility of pNET. Meanwhile, the similarity in the immunohistochemical profile indicates that the two tumors could potentially develop from the same origin.

## Introduction

Pancreatic neuroendocrine neoplasm (pNEN), initially called islet cell tumor, is a rare type of malignant tumor originating from pancreatic ductal epithelium with neuroendocrine differentiation. pNEN is estimated to constitute 1%–5% of all clinically detected pancreatic tumors, with an incidence of 0.8 cases per 100,000 (1). Owing to the increased number of detection methods and regular physical examinations, the incidence of pNEN has dramatically increased up to fourfold over the past half-century. The clinical features of pNEN have been reported to be widely heterogeneous, and it can be classified into functional and nonfunctional pNEN on the basis of clinical manifestation. It has been reported that approximately 90% of pNENs are nonfunctional, and high-quality imaging techniques have contributed to the exponential detection of nonfunctional pNENs (2). On the basis of histopathological findings, pNENs can be divided into two categories: well-differentiated pancreatic neuroendocrine tumors (pNETs) and poorly differentiated pancreatic neuroendocrine carcinomas (3). Well-differentiated pNETs have been confirmed to exhibit significantly better survival and a clearly different genomic background. Consistent with the broad heterogeneity, pNEN also exhibits a milieu of radiological appearance, varying tumor size and other morphological features, making it difficult to differentiate from other pancreatic neoplasms (4).

Solid pseudopapillary tumors (SPTs) are rare pancreatic neoplasms that account for 1%–2% of all pancreatic tumors. SPTs were originally described as papillary tumors of the pancreas in 1959, but the pathogenesis remains elusive with different hypotheses (5). Most cases tend to be asymptomatic, and no specific abnormality can be identified in clinical laboratory tests, including CA19-9 and serum amylase levels. In contrast to pNEN, SPT is widely believed to have low malignancy and exhibit a favorable prognosis after surgical resection. However, metastatic lesions can also occur in a small percentage of SPT cases, mostly liver metastasis (6). Typically, SPT patients present a large abdominal mass, and imaging features include internal hemorrhagic and cystic degeneration, but some rare radiological features can also be observed among SPT cases (4). Saleem et al. reported three cases of pancreatic lesions, which were presumptively diagnosed as SPT but turned out to be pNET in pathological diagnosis.

It is generally perceived that there are considerable disparities between pNETs and SPTs, such as the abundance of blood supply, solid or cystic pattern, and metastasis potential. However, considering the broad heterogeneity of pNETs and SPTs, the overlap of clinical manifestation and image appearance often confuses the discrimination. In clinical practice, we encounter a considerable number of pNET and SPT patients with difficult preoperative diagnosis and differentiation. Although several methods have been clinically employed, such as computed tomography (CT), endoscopic ultrasound (EUS), and positron emission tomography/computed tomography (PET/CT), and the analysis of clinical and radiological features has contributed to distinguishing pancreatic tumors, most prior studies have focused on a single radiological method rather than utilizing multimodality imaging (7, 8). Here, we comprehensively collected and analyzed the clinical data and multimodality imaging of 631 cases of pNET and SPT from our hospital from 2006 to 2021. Additionally, the morphological disparities and similarities indicated that there might exist some parallels in the genomic background and pathogenesis origin, for which we compared the immunohistochemical profiles of two tumors. To our knowledge, this is the first study to utilize different methods, including CT, magnetic resonance imaging (MRI), EUS, and PET/CT, to comprehensively investigate the disparity between pNETs and SPTs with the largest study samples among current studies.

## Materials and methods

### Study population

From March 2006 to January 2021, all patients with pathologically confirmed nonfunctional pNEN or SPT in our hospital were retrospectively reviewed. Given that the majority of SPTs are indolent and liver metastasis is rare, the selection of pNEN cases was limited to nonfunctional pNETs without distant metastasis when diagnosed. Patients with preoperative treatment and those with existing oncological events were also excluded. Finally, 354 cases of pNET and 277 cases of SPT were enrolled in our cohort. Approval from the Institutional Review Board was obtained before data were collected. Clinicopathological information was gathered from the electronic medical records and pathologic examination notes. This study was approved by the Ethics Committee of our hospital. The study was conducted in accordance with the Declaration of Helsinki (as revised in 2013). Written informed consent was obtained from all the study participants. Pathological diagnosis was confirmed by two senior pathologists independently.

### Procedure

Clinical information, including age, sex, clinical symptoms, tumor location, and tumor size, was collected from electronic medical records and pathological results (10.6084/m9.figshare.20073167). Multimodality imaging, including contrast-enhanced CT, MRI, EUS, and nuclear medicine imaging, was performed as previously described, and data were recollected through electronic medical systems (7, 9–11). Two experienced radiologists independently evaluated the variables of CT/MRI on a PACS workstation (GE Centricity PACS V 3.0, GE Healthcare, Milwaukee, WI, United States). Among our cohort, 259 (73.1%) pNET patients and 219 (79.1%) SPT patients underwent CT examinations in our hospital, mostly contrast-enhanced CT. In cases where only a small percentage of patients underwent MRI examinations, the imaging characteristics were mostly evaluated by CT images, and available MRI images were utilized to validate equivocal cases. The radiological features were evaluated through morphology, margin, solid and cystic pattern, enhancement intensity, multifocal lesion, calcification, pancreatic or biliary duct dilation, and lymph node (LN) enlargements. Notably, strong enhancement in the arterial phase or not was defined as the significant enhancement of a lesion over that of the peripheral pancreas. EUS examinations were performed by two experienced endoscopists who independently evaluated the EUS characteristics. Radiopharmaceuticals administered in nuclear medicine images included ^18^F-FDG, ^99m^Tc-HYNIC-TOC, and ^68^Ga-DOTATATE, and PET/CT images were independently analyzed by two nuclear medicine physicians. The radiological data were retrospectively evaluated with the reviewer blinded to the clinicopathological diagnosis except for sex, age, and clinical manifestation.

### Statistical analysis

The statistical analysis was performed using SPSS, version 24.0 (SPSS Inc., Chicago, IL, United States). Demographic and clinical characteristics were summarized as medians for continuous variables and percentages for categorical variables. Continuous data for the two tumor groups were compared using Student's *t*-test or the Wilcoxon signed rank test. Data of categorical variables were analyzed by the *χ*^2^ test or Fisher's exact test. Significant variables in the univariate analysis were summarized and underwent multivariate logistic regression analysis. A two-sided *P *< 0.05 was considered significant. Nomogram construction used a multivariate logistic model based on age, tumor size, strong enhancement intensity, calcification, and LN enlargement. R V.4.0.3 (R Foundation for Statistical Computing, Vienna, Austria) was used to construct and validate the nomogram.

## Results

### Clinical characteristics

The clinical characteristics of 631 cases are summarized in Table 1. The female patient percentage of pNET cases was slightly higher than that of male patients, while the female-to-male ratio among SPT cases was nearly 3:1. The average age of the SPT group was significantly younger than that of pNET group (35.3 vs. 53.3 years, *P* < 0.001), with more than 90% of SPT patients younger than 50 years. In contrast, those aged 50 years and older constituted up to 65% of the pNET group. Furthermore, SPT cases display a larger mean tumor size than pNET cases (4.8 vs. 2.8 cm, *P *< 0.001). Additionally, for patients younger than 18 years in the SPT group, the mean tumor size was 7.7 cm, ranging from 2.6 to 12 cm. In terms of tumor location, the pNET group exhibited a higher occurrence in the head than the SPT group (34.5% vs. 24.9%, *P *= 0.021), but both tumors displayed a higher tendency to occur in the pancreas body or tail. Most of the pNETs and SPTs shared similar clinical manifestations, with most cases being asymptomatic, and existing symptoms were mostly nonspecific, including abdominal pain, abdominal distension, discomfort, and lumbar and back pain. Serum tumor markers are summarized in Table 2, and no single marker reached a positive percentage of 20% (Table 2). Compared with pNET patients, SPT patients showed a higher potential to be positive for CA125 and CA242 (*P *= 0.039 and *P *= 0.058, respectively). All patients underwent surgical resection, and surgical procedures included pancreaticoduodenectomy (Whipple), distal pancreatectomy with splenectomy (DP), spleen-preserving distal pancreatectomy (SPDP), enucleation, total pancreatectomy resection, pylorus-preserving pancreaticoduodenectomy (PPPD), and central pancreatectomy. Surgical data are presented in Table 1, among which DP was the most common procedure and was performed in half of the cases of pNET and SPT (49.4% and 54.2%, respectively). Notably, the SPT group had a higher percentage of function-preserving surgery than the pNET group, including SPDP (9.4% vs. 5.1%), central pancreatectomy (6.9% vs. 3.7%), and PPPD (6.5% vs. 5.1%).

**Table 1:** Demographic data of pNET and SPT cases.

Characteristics	Number of cases (%)		*p* value
	pNET	SPT	
Gender			
Female	196 (55.4)	202 (72.9)	<0.001
Male	158 (44.6)	75 (27.1)	
Tumor location			
Head	122 (34.5)	69 (24.9)	0.021
Body/tail	227 (64.1)	206 (74.4)	
Others	5 (1.4)	2 (0.7)	
Symptoms			
Asymptomatic	238 (67.2)	184 (66.4)	0.865
Abdominal pain	49 (13.8)	52 (18.8)	
Abdominal distension	9 (2.5)	17 (6.1)	
Discomfort	34 (9.6)	16 (5.8)	
Lumbar and back pain	5 (1.4)	1 (0.4)	
Others	19 (5.4)	7 (2.5)	
Age (years)			<0.001
	53.4 ± 12.4	35.3 ± 10.9	
Tumor size (cm)			<0.001
	2.8 ± 1.9	4.8 ± 2.8	
Procedures			
Whipple	94 (26.6)	49 (17.7)	
DP	175 (49.4)	150 (54.2)	
SPDP	18 (5.1)	26 (9.4)	
Enucleation	29 (8.2)	10 (3.6)	
Central pancreatectomy	13 (3.7)	19 (6.9)	
PPPD	18 (5.1)	18 (6.5)	
Total pancreatectomy	6 (1.7)	3 (1.1)	
Other	1 (0.3)	2 (0.7)	

pNET, pancreatic neuroendocrine tumor; SPT, solid pseudopapillary tumor; DP, distal pancreatectomy with splenectomy; SPDP, spleen-preserving distal pancreatectomy; PPPD, pylorus-preserving pancreaticoduodenectomy.

**Table 2:** Serum tumor markers of pNET and SPT.

	Tumor marker	CA19-9	CA125	CA72-4	CA15-3	CA50	CA242	AFP	CEA	NSE	FERR
SPT	Total number	145	137	151	111	137	141	138	141	36	79
	Positive number (%)	16 (11)	10 (7.3)	25 (16.6)	5 (4.5)	5 (3.6)	11 (7.8)	0 (0)	5 (3.5)	3 (8.3)	9 (11.4)
pNET	Total number	321	318	319	252	316	317	317	320	147	112
	Positive number (%)	37 (11.5)	9 (2.8)	40 (12.5)	7 (2.8)	9 (2.8)	11 (3.5)	2 (0.6)	21 (6.6)	11 (7.5)	16 (14.3)
*p* value		1.0	0.039	0.254	0.524	0.768	0.058	0.121	0.105	1.0	0.665

pNET, pancreatic neuroendocrine tumor; SPT, solid pseudopapillary tumor; NSE, neuron-specific enolase.

### CT/MRI data

**Figure 1:**
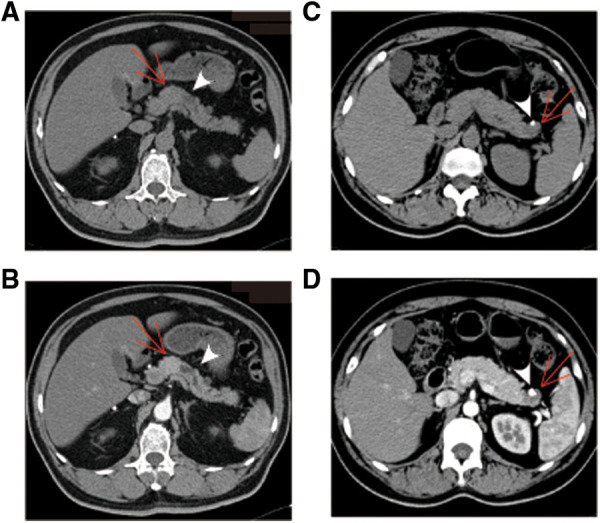
Typical CT images of pNET and SPT. (**A,B**) pNET in a 30-year-old man with no physical **discomfort. Axial unenhanced CT image demonstrated a regular and well-defined solid mass in the neck** of the pancreas [(**A**), arrow]. The mass was displayed as hyperattenuation and homogenous density in arterial phase of contrast-enhanced CT images (**B**). Apparent dilation of the distal pancreatic duct is observed (arrowhead). (**C,D**) SPT identified during physical examination in a 45-year-old woman. A cystic mass with low density and a well-defined margin was presented in the tail of the pancreas [(**C**), arrow]. The cystic portion was poorly enhanced in the arterial phase of contrast-enhanced CT images (**D**). Peripheral calcification was observed, with a size of 0.6 × 0.9 cm (arrowhead). pNET, pancreatic neuroendocrine tumor; CT, computed tomography; SPT, solid pseudopapillary tumor.

CT images have been an essential part of the early detection, triage, presurgical evaluation, and follow-up management of tumor patients. Typical CT images of pNETs and SPTs are displayed in Figure 1. As per the result summarized in Table 3, pNETs and SPTs showed no significant differences in morphologic regularity or margin clarity. In contrast, most pNETs are predominantly solid (71.5%), and cystic composition was not commonly observed. Meanwhile, approximately one-third of SPTs were predominantly cystic and 70% of SPTs contained cystic compositions. Additionally, most pNETs were obviously enhanced after contrast agent injection, while SPTs displayed scarce vascularization. Moreover, SPTs had a significantly higher tendency to be unevenly enhanced, corresponding to the more common internal hemorrhage and cystic components. Calcification was remarkedly observed in SPT, accounting for 37.3% of cases, compared to only 8.5% of pNET. In parallel with metastasis in the early stage, lymph node enlargement was significantly more common in pNET cases. Furthermore, pNETs tended to occur as multifocal lesions, and more duct dilation cases were observed.

**Table 3:** Radiological features of CT and/or MRI images.

Radiological features	Number of cases (%)		*p* value
	pNET	SPT	
Morphology			1.00
Regular	200 (76.9)	167 (77.0)	
Irregular	60 (23.1)	50 (23.0)	
Margin			0.363
Well-defined	211 (81.2)	168 (77.4)	
Ill-defined	49 (18.8)	49 (22.6)	
Solid and cystic pattern			<0.001
Predominantly solid	186 (71.5)	59 (27.2)	
Solid-cystic	38 (13.8)	80 (36.9)	
Predominantly cystic	36 (14.6)	78 (35.9)	
Enhancement intensity			<0.001
Minimal	27 (10.4)	25 (11.5)	
Weak	85 (32.8)	183 (84.3)	
Strong	147 (56.8)	9 (4.1)	
Enhancement pattern			<0.001
Homogeneous	118 (50.0)	31 (16.0)	
Heterogeneous	118 (50.0)	163 (84)	
Multifocal			0.001
Yes	11 (4.2)	0	
No	249 (95.8)	217 (100)	
Calcification			<0.001
Yes	22 (8.5)	81 (37.3)	
No	238 (91.5)	136 (61.8)	
Duct dilation			0.004
Yes	45 (17.3)	18 (8.3)	
No	215 (82.7)	199 (91.7)	
LN enlargement			<0.001
Yes	16 (6.2)	1 (0.5)	
No	244 (93.8)	216 (99.5)	

CT, computed tomography; MRI, magnetic resonance imaging; pNET, pancreatic neuroendocrine tumor; SPT, solid pseudopapillary tumor; LN, lymph node.

### EUS

**Figure 2:**
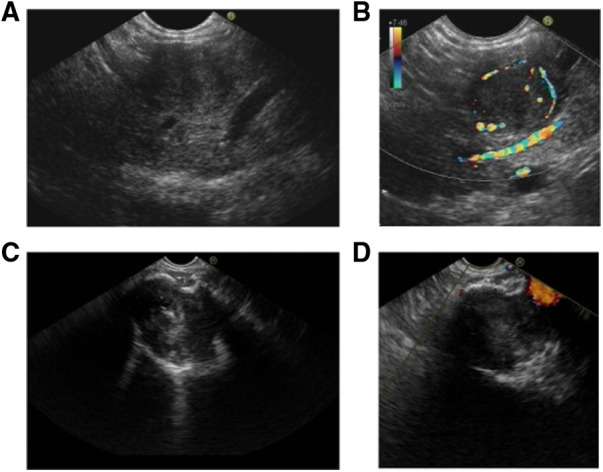
Characteristics of pNET and SPT in EUS. (**A,B**) pNET in a 54-year-old man with no physical discomfort. A homogeneous and hypoechoic lesion with a clear border was found in the pancreatic body. CDFI indicated the distinct peritumoral blood flow. (**C,D**) SPT identified in a 36-year-old woman during physical examination. A heterogeneous and hypoechoic lesion with a clear border was identified in the pancreatic neck. CDFI showed no significant blood flow signal. Hyperechoic calcification was observed in the lesion. pNET, pancreatic neuroendocrine tumor; EUS, endoscopic ultrasound; SPT, solid pseudopapillary tumor; CDFI, color Doppler flow imaging.

EUS has been reported to be feasible in clarifying equivocal images on CT or MRI, and fine needle aspiration can provide pathological evidence before clinical treatment. We defined the characteristics of EUS in five aspects, including echo intensity, homogeneity, regularity of shape, vascularization, and margin clarity (Table 4). Ninety-five (26.8%) pNET patients and 26 (9.4%) SPT patients received EUS examinations. In line with the CT findings, both shape and margin shared a higher similarity between pNETs and SPTs. Additionally, the echo pattern of SPTs was more likely to be heterogeneous than that of pNETs (96.2% vs. 53.7%). Likewise, pNET lesions displayed markedly higher vascularization, while most SPT lesions were hypovascular (Figure 2). However, the intensity of echo was similar between pNETs and SPTs, as most cases of both tumors were hypoechoic on EUS examination.

**Table 4:** EUS and PET/CT characteristics of pNET and SPT.

Characteristics	Number of cases (%)		*p* value
	pNET	SPT	
EUS			
Regular shape	71 (72.4)	18 (66.7)	0.633
Clear boundary	92 (95.8%)	23 (85.2)	0.123
Hypoechoic	87 (91.6)	26 (96.3)	0.069
Homogenous echo	51 (46.3)	1 (3.8)	<0.001
Hypervascularization	68 (72.3)	2 (8.7)	<0.001
PET/CT			
^18^F-FDG			0.01
Positive	22/36	13/13	
Negative	14/36	0/13	
SUVmax	4.4 ± 5.4	5.0 ± 2.0	0.619
^99m^Tc-TOC			<0.001
Positive	70/86	0/10	
Negative	16/86	10/10	
^68^Ga-TATE			—
Positive	19/20	—	
Negative	1/20	—	

EUS, endoscopic ultrasound; PET/CT, positron emission tomography/computed tomography; pNET, pancreatic neuroendocrine tumor; SPT, solid pseudopapillary tumor; SUV, standardized uptake value.

### PET/CT

**Figure 3:**
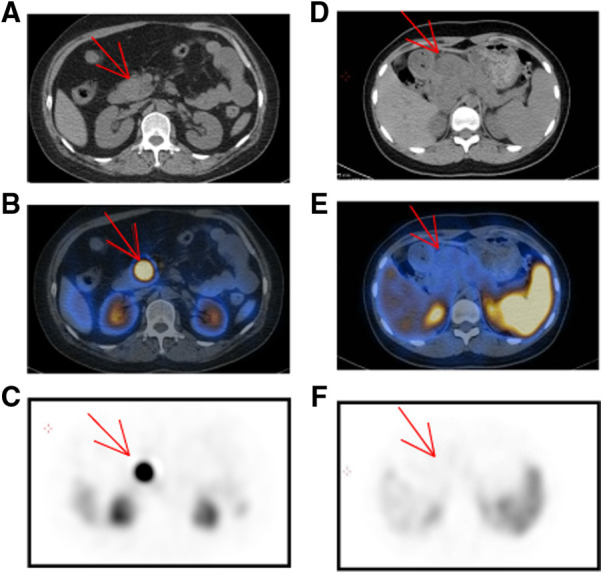
Characteristics of pNET and SPT in ^99m^Tc-HYNIC-TOC PET/CT. (**A–C**) A 47-year-old woman was suspected of pNET in the pancreatic head. Upon noncontrast CT examination, a predominant solid and regular shape mass was observed [(**A**), arrow]. In the ^99m^Tc-HYNIC-TOC PET/CT fusion images, there was intense activity in the pancreatic head [(**B**), arrow]. (**D–F**) SPT in a 14-year-old girl with abdominal pain. Upon unenhanced CT examination, a 5.8 × 5.2 cm mass was presented in the pancreatic head with an ill-defined margin and low density [(**D**), arrow]. In the ^99m^Tc-HYNIC-TOC PET/CT fusion images, there was no increased activity in the corresponding location with CT and peripheral tissue was apparently squeezed by the pancreatic mass. pNET, pancreatic neuroendocrine tumor; CT, computed tomography; PET, positron emission tomography; SPT, solid pseudopapillary tumor.

On the basis of tumor function and metabolism intensity, PET/CT has been emerging as a well-established technique in diagnosing malignant tumors. ^18^F-FDG is the most commonly used radiopharmaceutical in tumor detection. Additionally, adequate expression of somatostatin receptors in pNET facilitates nuclear medicine imaging with somatostatin analogs, such as ^99m^Tc-HYNIC-TOC and ^68^Ga-DOTATATE. In our cohort, 50 patients received ^18^F-FDG PET/CT, including 36 pNET cases and 13 SPT cases (Table 4). Interestingly, all 13 SPT patients exhibited increased FDG uptake to different degrees, while FDG uptake was only identified in 23 of 36 pNET patients (*P *= 0.01). However, the standardized uptake value (SUV) did not show a significant difference between the two lesions. In contrast, somatostatin receptor scintigraphy (SRS) showed a significant specificity in distinguishing pNETs from SPTs, as 10 SPT cases were all negative on ^99m^Tc-HYNIC-TOC PET/CT, while 70 of 86 pNET lesions were positive (Figure 3). Additionally, 20 pNET patients underwent ^68^Ga-DOTATATE PET/CT, and 19 patients showed increased ^68^Ga-DOTATATE uptake. In conclusion, SRS displays a higher specificity in distinguishing pNET and SPT than ^18^F-FDG PET/CT.

### Pathological features

The comprehensive pathological profile of pNET and SPT is summarized in Table 5. In line with higher malignancy, pNETs displayed significantly more vascular invasion and LN metastasis under microscopy, except for perineural invasion and peripheral aggression. Additionally, the Ki-67 immunolabeling index was markedly higher in pNETs than that in SPTs, with mean Ki-67 index values of 4.21% and 2.46%, respectively. To investigate the immunohistochemical profile of the two tumors, 16 markers were selected, including the neuroendocrine markers synaptophysin (Syn), neuron-specific enolase (NSE), chromaffin granule protein A (CgA), and CD56; somatostatin receptor 2 (SSTR2); classically mutated genes in the two tumors including *β*-catenin, ATRX, and DAXX; and pancreatic neoplasms markers including trypsin, chymotrypsin, vimentin, and CD10. A significant difference was observed in most of these markers except for NSE, ATRX, DAXX, trypsin, and chymotrypsin. Nevertheless, the expression of these markers overlapped widely in the two tumors. Strong immunolabeling of NSE and SSTR2, combined with weak expression of CD10, could be a specific marker for pNET. Notably, in pNET cases, the loss of immunolabeling for ATRX and DAXX (8.3% and 9.1%, respectively) was markedly less than in a previous report, which could result from the selection of nonmetastatic pNET cases (12, 13). Interestingly, positive immunolabeling of neuroendocrine markers was widely observed in SPT cases. A total of 82.9% and 95% of SPT lesions exhibited positive staining for Syn and NSE, respectively. Additionally, SSTR2-positive immunolabeling was identified in 6 of 49 SPT cases. The similarities in immunohistochemical profiles highlight the possibility that pNETs and SPTs could be generated from the same origin.

**Table 5:** Pathological profiles of pNET and SPT.

Characteristics	Total number (pNET/SPT)	Number of positive cases (%)		*p* value
		pNET	SPT	
Peripheral aggression	354/277	25 (7.1)	11 (4.0)	0.119
Vascular invasion	354/277	61 (17.2)	5 (1.8)	<0.001
LN metastasis	332/252	43 (13.0)	1 (0.4)	<0.001
Perineural invasion	354/277	36 (10.2)	22 (7.9)	0.405
ki67 (%)	345/229			<0.001
<3		182 (52.8)	162 (70.7)	
3–10		121 (35.1)	61 (26.6)	
≥10		42 (12.2)	6 (2.6)	
Syn	347/234	345 (99.4)	194 (82.9)	<0.001
*β*-catenin	278/254	263 (94.6)	251 (98.8)	0.008
CgA	343/234	334 (97.4)	19 (8.1)	<0.001
NSE	19/20	17 (89.5)	19 (95.0)	0.605
AE1/AE3	325/238	321 (98.8)	185 (77.7)	<0.001
SSTR2	200/49	193 (96.5)	6 (12.2)	<0.001
ATRX	96/7	88 (91.7)	7 (100)	1.0
DAXX	88/7	80 (90.9)	7 (100)	1.0
Vim	33/73	13 (39.4)	72 (98.6)	<0.001
CD99	133/181	94 (70.7)	136 (75.1)	0.439
PR	131/224	90 (68.7)	216 (96.4)	<0.001
CD56	150/103	113 (75.3)	97 (94.2)	<0.001
CD10	103/182	19 (18.4)	176 (96.7)	<0.001
Trypsin	153/91	2 (1.3)	0 (0)	0.530
Chymotrypsin	73/36	73 (100)	36 (100)	—

pNET, pancreatic neuroendocrine tumor; SPT, solid pseudopapillary tumor; LN, lymph node; NSE, neuron-specific enolase.

## Multivariate analysis and nomogram construction

**Figure 4:**
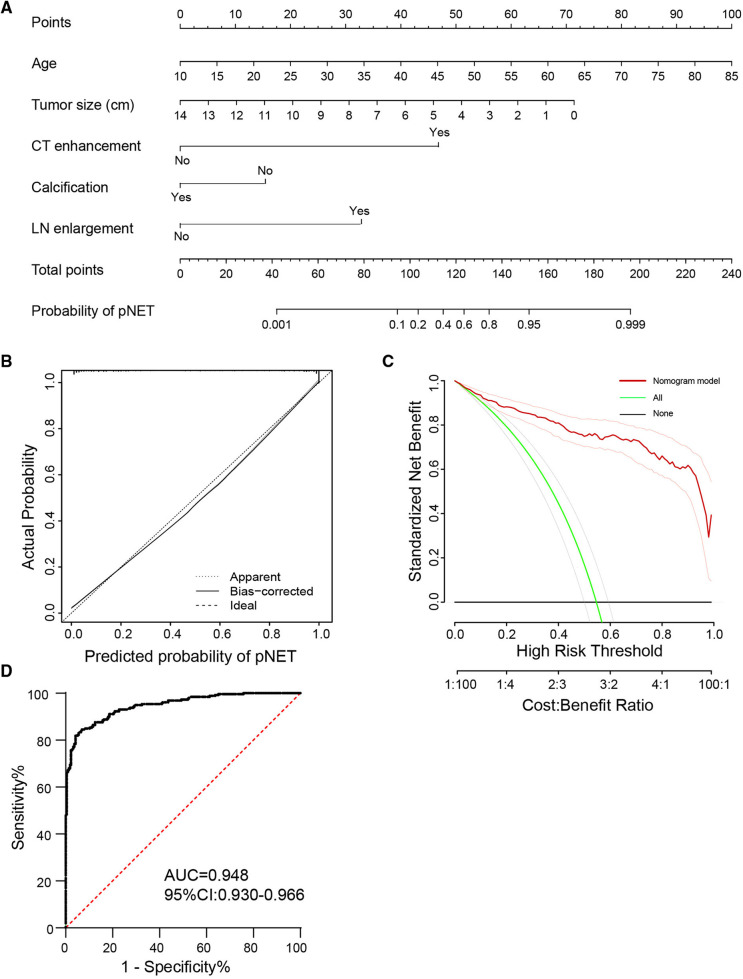
Nomogram and ROC curve of distinguishing pNET from SPT by five clinicopathological features. (**A**) A nomogram incorporating the presence of age, tumor size, strong enhancement intensity, calcification, and LN enlargement. (**B,C**) Calibration curve and decision curve analysis to distinguish two tumors using the nomogram (**A**). (**D**) ROC curve of the model in differentiating pNET and SPT. ROC, receiver operator curve; LN, lymph node; pNET, pancreatic neuroendocrine tumor; SPT, solid pseudopapillary tumor.

Essentially, CT and serum tumor marker examinations are two basic methods to evaluate tumor properties and guide clinical treatment. To better differentiate the two tumors in clinical practice, we further constructed a logistic model to predict the tumor diagnosis. Patients with contrast-enhanced CT results were selected for the new cohort, including 259 pNET and 217 SPT patients. Features of demographic information, contrast-enhanced CT, and serum tumor markers with statistical significance in univariate analysis were evaluated in the logistic regression analysis (Table 6). Age, tumor size, strong enhancement intensity, calcification, and LN enlargement were confirmed to be crucial features in distinguishing pNETs and SPTs. Based on the multivariate logistic model, we constructed a nomogram, incorporating age, tumor size, strong enhancement intensity, calcification, and LN enlargement to distinguish pNETs and SPTs (Figure 4A). The calibration plots showed good agreement between the predicted and actual probabilities, and decision curve analysis displays strong efficacy (Figures 4B,C). The area under the receiver operator curve (AUROC) was used to test the accuracy of the model and displayed an AUC of 0.948 (95% CI = 0.930–0.966), which was better than that of the model using a single examination (Figure 4D, Supplementary Figure S1).

**Table 6:** Multivariate analysis of demographic and radiological features.

Variable	*p* value	OR	95% CI
Age	<0.001	1.12	1.08–1.16
Tumor size	0.006	0.72	0.57–0.91
Strong enhancement intensity	0.008	28.1	2.44–324
Calcification	0.005	0.28	0.12–0.69
LN enlargement	0.007	62.75	3.06–1286

OR, odds ratio; CI, confidence interval; LN, lymph node.

### Comparison of SPTs with different grade pNETs

To further investigate the difference between SPTs and pNETs, we divided pNETs into two groups, grade 1 (G1) and grade 2/3 (G2/3), and compared the radiological and pathological features between the pNET G1, pNET G2/3, and SPT groups (Table 7). We first compared the pNET G1 and G2/3 group with the SPT group, and most characteristics were significantly different in both G1 and G2/3. Notably, we identified that G1 and SPT shared similar rates of duct dilation and LN enlargement. Additionally, G2 displayed a similar positive rate in ^18^F-FDG PET/CT to the SPT group. Later, we evaluated the disparity between G1 and G2/3. Compared to pNET G1, G2/3 displayed a remarkably larger tumor size (3.5 vs. 2.2 cm) and pathological progression in vascular and LN invasion. Regarding morphology, G2/3 was more likely to possess heterogeneous patterns in both contrast-enhanced CT and EUS. However, although G2/3 tended to exhibit lower vascularization, this difference was not significant on either contrast-enhanced CT or EUS (59.2% vs. 53.9%, 77.4% vs. 67.5%, respectively). Additionally, the positive rate of ^18^F-FDG PET/CT was almost doubled in G2/3 compared with that in G1 (44.4% vs. 82.4%, *p *< 0.05).

**Table 7:** Comparison of SPT with different grade pNET.

Characteristics	Percentage of cases/mean (*p* value)		
	pNET (G1) vs. SPT	pNET (G2/3) vs. SPT	pNET (G1) vs. pNET (G2/3)
Sex (female)	54.6% vs. 72.9%****	56.5% vs. 72.9%****	54.6% vs. 56.5% (ns)
Age (years)	53.3 vs. 35.3****	53.6 vs. 35.3****	53.3 vs. 53.6 (ns)
Tumor size (cm)	2.2 vs. 4.8****	3.5 vs. 4.8****	2.2 vs. 3.5****
Solid and cystic pattern (predominantly solid)^a^	72.5% vs. 27.2%****	69.8% vs. 27.2%****	72.5% vs. 69.8% (ns)
Enhancement intensity (strong)^a^	59.2% vs. 4.1%****	53.9% vs. 4.1%****	59.2% vs. 53.9% (ns)
Enhancement pattern (homogeneous)^a^	55.9% vs. 16.0%****	42.1% vs. 16.0%****	55.9% vs. 42.1%*
Multifocal (yes)^a^	4.9% vs. 0**	3.4% vs. 0*	4.9% vs. 3.4% (ns)
Calcification (yes)^a^	7.0% vs. 38.2%****	10.3% vs. 38.2%****	7.0% vs. 10.3% (ns)
Duct dilation (yes)^a^	13.4% vs. 8.2% (ns)	22.2% vs. 8.2%****	13.4% vs. 22.2% (ns)
LN enlargement (yes)^a^	2.1% vs. 0.5% (ns)	11.1% vs. 0.5%****	2.1% vs. 11.1%**
Homogenous echo (yes)^b^	54.7% vs. 3.8%****	34.1% vs. 3.8%**	54.7% vs. 34.1%*
Hypervascularization (yes)^b^	77.4% vs. 8.7%****	67.5% vs. 8.7%****	77.4% vs. 67.5% (ns)
^18^F-FDG PET/CT (positive)	44.4% vs. 100%**	82.4% vs. 100% (ns)	44.4% vs. 82.4%*
^99m^Tc-TOC PET/CT (positive)	83.0% vs. 20.0%****	81.3% vs. 20.0%***	82.4% vs. 81.3% (ns)
Vascular invasion^c^	7.7% vs. 1.8%**	28.0% vs. 1.8%****	7.7% vs. 28.0%****
LN metastasis^c^	7.8% vs. 0.4%****	18.4% vs. 0.4%****	7.8% vs. 18.4%**

* <0.05; **<0.01; ***<0.001; ****< 0.0001.

^a^ Examination under contrast-enhanced CT.

^b^ Examination under EUS.

^c^ Pathological examination.

EUS, endoscopic ultrasound; PET/CT, positron emission tomography/computed tomography; pNET, pancreatic neuroendocrine tumor; SPT, solid pseudopapillary tumor.

## Discussion

Neuroendocrine tumors (NETs) are a group of heterogeneous neoplasms, and their incidence has increased approximately sevenfold over the past half-century. The bronchopulmonary tree and gastroenteropancreatic tract are two of the most common locations of NETs. The origin of NET has been a long-debated question, and it is generally believed to arise from the secretory cells of the diffuse neuroendocrine system. Primarily, pNETs are derived from the pancreatic ductal epithelium with neuroendocrine differentiation. The wide heterogeneity results in a complex grading system of pNETs. Nevertheless, the categories of pNETs remain elusive. Recently, next-generation sequencing has identified molecular drivers of pNET, involved in chromatin remodeling, DNA damage repair, telomere maintenance, and mTOR signaling pathways (12). MEN1, ATXX, DAXX, PTEN, and SETD2 are the top mutated genes in nonfunctional pNETs. However, a remarkable difference in genetic background exists between well-differentiated pNETs and poorly differentiated pNETs, raising doubt regarding whether all pNETs share the same origin or evolutionary path (14). Additionally, the morphology of pNETs has recently been revealed to be variable, as cystic pNETs could be a subgroup since they display indolent biological behavior and favorable prognosis (15). Additionally, the stromal and immune microenvironment of cystic pNETs is distinct from that of solid pNETs (16). The wide heterogeneity has made it difficult to identify and differentiate pNETs from other neoplasms.

The discrimination of pNETs and SPTs has been another long-debated question. The overlap of morphology and biological behavior makes them display similar features, such as cystic components, well-defined margins, regular shapes, and rich or poor vascularity. In clinical practice, we have observed a considerable number of cases with an equivocal preoperational diagnosis of pNET or SPT. In this case, we carried out an observational study to evaluate the clinical, radiological, and pathological differences between the two tumors. Nearly all of the SPT cases in our center are indolent lesions without liver metastasis, while liver metastasis is frequently detected in pNET cases. Therefore, we focused on the comparison of SPT cases with nonfunctional and localized pNET cases. Univariate analysis of clinical characteristics showed that pNETs tend to occur in older women with smaller tumor size, while SPTs tend to occur in young women aged 20–40 years. Most serum tumor markers were negative in both tumors but the positive rate of CA125/242 was much higher in SPT patients. Although pNETs and SPTs have significant differences in solid-cystic patterns and other radiological features, the multivariate analysis identified tumor size, age, enhancement intensity, calcification, and LN enlargement as statistically significant variables. A logistic regression model of these five variables was established with a sensitivity of 86.4% and a specificity of 87.2%, and the AUROC confirmed a favorable efficacy in distinguishing the two tumors.

Similar to the CT results, hypervascularization remained the most helpful feature in distinguishing the two tumors. Furthermore, PET/CT with different radiopharmaceuticals was widely used in equivocal cases, but we found that the efficacy of ^18^F-FDG PET/CT might be lower than that of the SRS. Most pNETs were positive on ^99m^Tc-HYNIC-TOC and ^68^Ga-DOTATATE PET/CT, and SPTs were not sensitive to SRS. Former reports regarding the use of PET/CT in SPT cases are scarce, and most of these studies mainly focus on ^18^F-FDG PET/CT. Yoo et al. reported that ^68^Ga-DOTA-TOC PET/CT was highly valued in assessing suspected pancreatic neuroendocrine neoplasms (15). Furthermore, ^68^Ga PET/CT has shown promising results in identifying occult lesions and helping with follow-up management. In our study, all 10 cases of SPT were negative on ^99m^Tc-HYNIC-TOC PET/CT, confirming that SRS could be a feasible method to exclude suspected SPT cases in clinical practice.

First described by Frantz in 1959, sporadic SPT cases have been reported from different institutions. However, the genetic background and origin of SPTs remain elusive. In contrast to pancreatic ductal adenocarcinoma and pNETs, SPTs are characterized by Wnt-*β*-catenin signaling pathway alterations, and most SPT cases harbor *β*-catenin gain-of-function mutations (16). However, the histopathological profiles of SPT cases have been investigated in many studies, and the expression of neuroendocrine markers has been widely observed. Therefore, it is commonly believed that SPTs exhibit focal endocrine cell differentiation (17, 18). Additionally, SPTs could originate from undifferentiated cells with multiple differential potentials. This hypothesis has been validated by electron microscopy as neurosecretory granules have been ultrastructurally observed in SPT cells (19, 20). In some rare cases, the SPT lesion may predominantly consist of endocrine cells (21). However, most of this evidence depends on the histopathological results, while the genetic background has seldom been investigated. Further research into the mutational profile by whole-genome sequencing might be needed to uncover the origin and evolutionary path of SPTs.

Considering the nature of this retrospective study, there were several limitations of this research. First, as clinical data were mainly collected in our single center, radiological images of some cases were unavailable, and future prospective studies could avoid this selection bias. In addition, even though a large number of clinical cases were enrolled, our study was predominantly limited to clinical data, but the similarities and disparities of pNET and SPT were not investigated *per se*. Studies performing high-throughput sequencing of pNET and SPT remain scarce to date, and uncovering the internal and external relationships of the two tumors is of high necessity in future studies.

## Data Availability

The datasets presented in this study can be found in online repositories. The names of the repository/repositories and accession number(s) can be found in the article/Supplementary Material.
