# Supplementary figures and images for: Multimodality imaging differentiation of pancreatic neuroendocrine tumors and solid pseudopapillary tumors with a nomogram model: A large single-center study
Source: Front Surg. 2022 Oct 6;9:970178. doi: 10.3389/fsurg.2022.970178 (PMC9583874; doi:10.3389/fsurg.2022.970178)

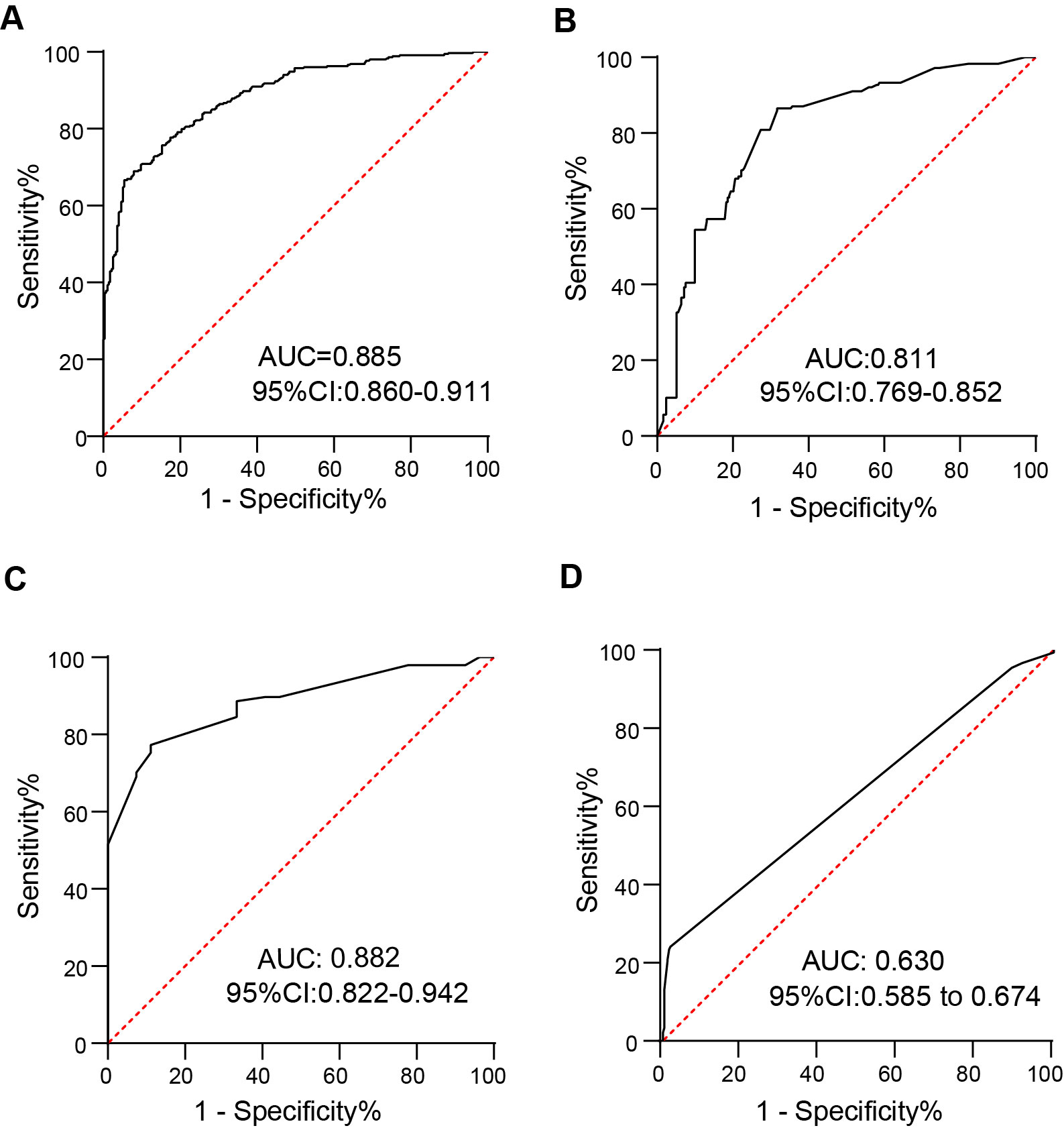

Supplement: Supplementary file 1 [file Image1.jpeg]
